# Elucidating the Role of CD84 and AHR in Modulation of LPS-Induced Cytokines Production by Cruciferous Vegetable-Derived Compounds Indole-3-Carbinol and 3,3′-Diindolylmethane

**DOI:** 10.3390/ijms19020339

**Published:** 2018-01-24

**Authors:** Thomas T. Y. Wang, Quynhchi Pham, Young S. Kim

**Affiliations:** 1 Diet, Genomics and Immunology Lab, Beltsville Human Nutrition Research Center, The Agricultural Research Service (ARS), United States Department of Agriculture (USDA), Beltsville, MD 20705, USA; Quynhchi.Pham@ars.usda.gov; 2 Nutritional Science Research Group, Division of Cancer Prevention, National Cancer Institute, Rockville, MD 20850, USA

**Keywords:** aryl hydrocarbon receptor, CD84, indole-3-carbinol, 3,3′-diindolylmethane, IL-1β, macrophage

## Abstract

Modulation of the immune system by cancer protective food bioactives has preventive and therapeutic importance in prostate cancer, but the mechanisms remain largely unclear. The current study tests the hypothesis that the diet-derived cancer protective compounds, indole-3-carbinol (I3C) and 3,3′-diindolylmethane (DIM), affect the tumor microenvironment by regulation of inflammatory responses in monocytes and macrophages. We also ask whether I3C and DIM act through the aryl hydrocarbon (AHR)-dependent pathway or the signaling lymphocyte activation molecule (SLAM) family protein CD84-mediated pathway. The effect of I3C and DIM was examined using the human THP-1 monocytic cell in its un-differentiated (monocyte) and differentiated (macrophage) state. We observed that I3C and DIM inhibited lipopolysaccharide (LPS) induction of IL-1β mRNA and protein in the monocyte form but not the macrophage form of THP-1. Interestingly, CD84 mRNA but not protein was inhibited by I3C and DIM. AHR siRNA knockdown experiments confirmed that the inhibitory effects of I3C and DIM on IL-1β as well as CD84 mRNA are regulated through AHR-mediated pathways. Additionally, the AHR ligand appeared to differentially regulate other LPS-induced cytokines expression. Hence, cross-talk between AHR and inflammation-mediated pathways, but not CD84-mediated pathways, in monocytes but not macrophages may contribute to the modulation of tumor environments by I3C and DIM in prostate cancer.

## 1. Introduction

During prostate tumor progression, interactions occur between cancer cells and innate immune cells, including monocytes and macrophages [1,2]. These interactions create a complex tumor microenvironment that appears to be critical to both the initiation and maintenance of prostate tumorigenesis [1,2,3,4]. Monocytes and macrophages respond to pathogens and provide a primary defense that ultimately leads to pathogen clearance [5,6]. These cells are important for innate immune responses, producing pro-inflammatory cytokines such as IL-1β, and contributing to Toll-like receptors (TLR)-mediated tuning of adaptive immune responses [7,8]. The TLRs are a family of well-known pattern recognition receptors (PRRs) in both monocytes and macrophages [9], and are known to induce inflammatory responses that converge at the nuclear factor NF-κB to stimulate the release of cytokines such as IL-1β and interleukin-6 (IL-6) [9,10,11]. IL-1β is known to be induced by inflammatory TLR ligands such as the bacterial lipopolysaccharide (LPS) in monocytes and macrophages [11]. Moreover, IL-1β, acting together with vascular endothelial growth factor (VEGF), appeared to be a major mediator in the tumor microenvironment and plays a crucial role in mounting and maintaining tumor-mediated angiogenesis [12,13]. Compounds that block IL-1β-mediated pathways could potentially attenuate tumor progression. 

The human CD84 is a member of the signaling lymphocyte activation molecule (SLAM) family protein (also known as SLAM5) that is expressed in a variety of immune cells, including monocytes and macrophages [14,15]. The role and regulation of CD84 in monocytes and macrophages are less clear. However, a recent study by Sintes et al. [14], using siRNA knockout of CD84, reported a role for CD84 in modulating LPS induction of inflammatory cytokines IL-6 and Tumor Necrosis Factor-α (TNF-α) with no apparent effect on C-C Motif Chemokine Ligand 2/Monocyte Chemoattractant Protein-1 (CCL2/MCP-1) production in a mouse bone marrow-derived macrophage. These reported results support a role for CD84 in the regulation of immune responses, but the effects of CD84 on LPS induction of IL-1β are not known and warrant further elucidation.

Recent studies also implicate the aryl hydrocarbon receptor (AHR), a critical transcription factor in xenobiotic metabolism, participating in a cross talk with both innate and adaptive immune responses in various immune cell types, including lymphocytes and antigen-presenting cells (APCs) [16,17]. Kimura et al. [18] reported that LPS-induced production of IL-6, TNF-α and IL-12 is augmented in Ahr−/− peritoneal macrophages compared with wild type cells. Moreover, compared with wild type mice, Ahr−/− mice are more sensitive to LPS [18,19]. Consistent with these findings, bone marrow-derived macrophages from Ahr−/− mice produce increased levels of IL-1β compared to wild type cells [19]. A recent study by Masuda et al. [20] also reported that overproduction of histamine in Ahr−/− peritoneal macrophages drives IL-6 expression. Consequently, in addition to its classical role as a toxicological signal mediator, AHR may also regulate immune responses. AHR ligand, such as those commonly found in the diet, may thus contribute to the attenuation of inflammatory responses and modulate the tumor microenvironment. 

Diet-derived compounds, due to their perceived safety, are often sought out as the prime candidate for cancer prevention. Indole-3-carbinol (I3C) is one such putative diet-derived cancer preventive compound that is derived from the hydrolysis of glucobrassicin in cruciferous vegetables such as broccoli, cabbage and cauliflower [21]. I3C can be converted to the dimeric form 3,3′-diindolylmethane (DIM) in the acidic pH of the stomach [22,23]. Previous studies, including our own, have shown that I3C and DIM inhibit prostate cancer cell growth in vitro and in vivo [24,25,26,27,28]. In vitro, both I3C and DIM appear to be pleotropic and can exert their effects through multiple pathways which include modulation of androgen-dependent pathways and the AHR mediated pathway [23,24]. Previously, we have shown that I3C and DIM, through androgen-dependent pathways, modulate the interaction between prostate cancer cells and macrophages by regulating the production of chemokine CCL2 by prostate cancer cells [29]. CCL2 is a pro-inflammatory chemokine that attracts monocytes to tumor sites [30]. Inhibition of CCL2 by I3C or DIM in the prostate cancer cells may indirectly minimize inflammation in tumor sites, influence tumor microenvironment and prevent prostate cancer development. The effects of I3C and DIM on immune cells such as monocytes and macrophages are less clear. To test the effects of I3C and DIM on monocyte/macrophage, we selected the human THP-1, a monocytic cell line derived from an acute monocytic leukemia patient. THP-1 is a monocytic cell line that can be differentiated into macrophage. Additionally, we have also shown that THP-1 can be attracted by cancer cells [29].

In a preliminary study using in-silico approach, we identified multiple putative AHR responsive elements in the promoter region of CD84 [31]. Based on this information, as well as the existing literature on AHR and CD84-mediated immune responses [14,15,16,17,18,19,20], we initially hypothesized that the AHR ligands I3C and/or DIM, through activation of AHR, may downregulate CD84 and subsequently inhibit LPS induction of IL-1β in both monocytes and macrophages. However, our results demonstrate a role for AHR but not CD84 in inhibiting IL-1β production in monocytes by I3C and DIM. Moreover, the effects of these two compounds on other LPS-stimulated cytokine production in macrophages were limited. In addition, we also observed a disconnect between the transcriptional and translational regulation of CD84 by I3C and DIM.

## 2. Results

### 2.1. Effects of Indole-3-Carbinol (I3C) and 3,3′-Diindolylmethane (DIM) on Lipopolysaccharide (LPS) Induction of IL-1β in THP-1 Monocytic Cells

**Figure 1:**
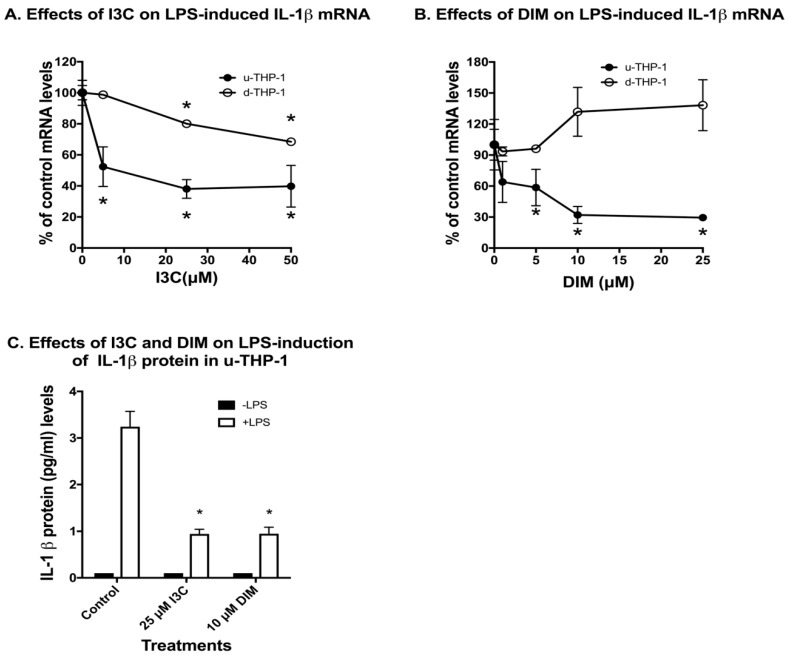
Effects of indole-3-carbinol (I3C) and 3,3′-diindolylmethane (DIM) on lipopolysaccharide (LPS) induction of IL-1β mRNA and protein levels in THP-1 cells. THP-1 cells were differentiated using phorbol 12-myristate 13-acetate (PMA) as described in Materials and Methods. U-THP-1 and d-THP-1 cells were treated with or without I3C or DIM as described in Materials and Methods. After treatment with the compounds, cells were exposed to LPS for 2 h and total RNA isolated and gene expression determined using RT-PCR. The treatment results were normalized to LPS-induced IL-1β mRNA level in the absence of compounds and expressed as % control (*n* = 3, mean ± standard deviation (SD)). For determination of IL-1β protein, media were collected after 5 h and IL-1β protein was determined using ELISA as described in Materials and Methods. (**A**) Effects of I3C on LPS induction of IL-1β mRNA in u-THP-1 and d-THP-1; (**B**) Effects of DIM on LPS induction of IL-1β mRNA in u-THP-1 and d-THP-1; (**C**) Effects of I3C and DIM on IL-1β protein secretion in u-THP-1. Results are expressed as mean ± SD (*n* = 3). * indicates significant difference from vehicle control at *p* ≤ 0.05.

Using the human THP-1 cells as a model, we first examined the effects of I3C and DIM on LPS induction of IL-1β mRNA expression. Treatment of un-differentiated THP-1 (u-THP-1) monocytes with I3C or DIM led to a concentration-dependent inhibition of LPS-induced increase in IL-1β mRNA levels (Figure 1A,B).

In u-THP-1, both I3C and DIM, at 5 µM, significantly inhibited LPS-induced IL-1β mRNA levels. However, in PMA-differentiated (d-THP-1) cells, we found that I3C inhibited IL-1β mRNA only at concentrations ≥ 25 µM (Figure 1A). A similar inhibitory effect was not observed for DIM at higher concentrations in d-THP-1 (Figure 1B). At the protein level, exposure of u-THP-1 cells to LPS led to an increase in IL-1β protein in the media and treatment with I3C (25 µM) and DIM (10 µM) significantly attenuated LPS-induced release of IL-1β protein in the media (Figure 1C).

### 2.2. Effects of I3C and DIM on CD84 and Aryl Hydrocarbon (AHR)-Responsive Gene CYP1A1/B1 mRNA Levels in THP-1 Cells

**Figure 2:**
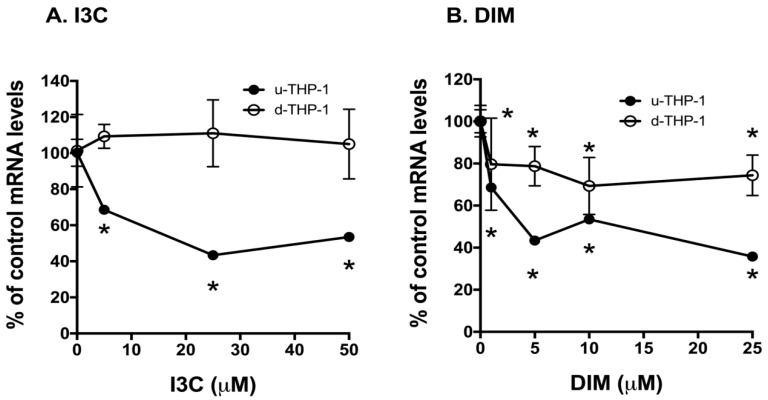
Effects of I3C, DIM on CD84 mRNA levels in THP-1 cells. THP-1 cells were differentiated using PMA as described in Materials and Methods. U-THP-1 and d-THP-1 cells were treated with or without I3C or DIM as described in Materials and Methods. After treatment with the compounds, cells were exposed to LPS for 2 h, total RNA isolated and CD84 mRNA expression determined using RT-PCR. The treatment results were normalized to LPS-induced IL-1β mRNA level in the absence of compounds and expressed as % control (*n* = 3, mean ± SD). (**A**) Effect of I3C; (**B**) Effect of DIM. * indicates significant difference from vehicle control at *p* ≤ 0.05.

To test the potential role of CD84 in mediating the effects of I3C and DIM on IL-1β, we first examined the effects of I3C and DIM on CD84 mRNA expression in THP-1 cells. Exposure of u-THP-1 cells to I3C and DIM led to a concentration-dependent inhibition of CD84 mRNA levels (Figure 2A,B).

**Figure 3:**
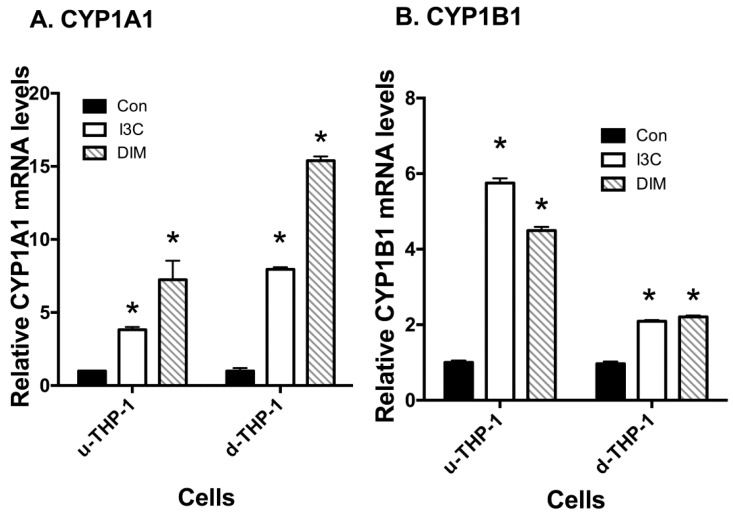
Effects of I3C and DIM on CYP1A1/B1 mRNA levels in THP-1 cells. THP-1 cells were differentiated using PMA as described in Materials and Methods. U-THP-1 and d-THP-1 cells were treated with or without I3C or DIM as described in Materials and Methods. After treatment with the compounds, total RNA was isolated and gene expression was determined using RT-PCR. (**A**) Effects on CYP1A1; (**B**) Effects on CYP1B1. Results are expressed as mean ± SD (*n* = 3). * indicates significant difference from vehicle control at *p* ≤ 0.05.

I3C and DIM significantly inhibited CD84 mRNA levels at 5 and 1 µM, respectively, in u-THP-1. Also, the effects of I3C and DIM on CD84 mRNA in d-THP-1 appeared to be minimal. To examine the role of AHR, AHR-inducible genes CYP1A1/B1 mRNA in u- and d-THP-1 cells were determined. As expected, I3C and DIM treatment induced the AHR-dependent genes CYP1A1/B1 mRNA levels (Figure 3A,B).

### 2.3. Effects of I3C, DIM and PMA on CD84 Protein in THP-1 Cells

**Figure 4:**
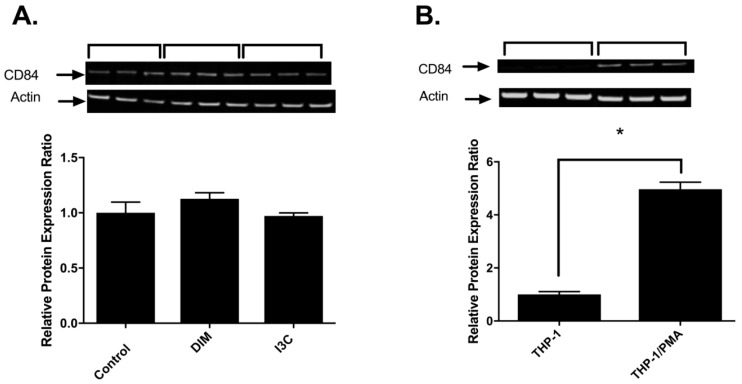

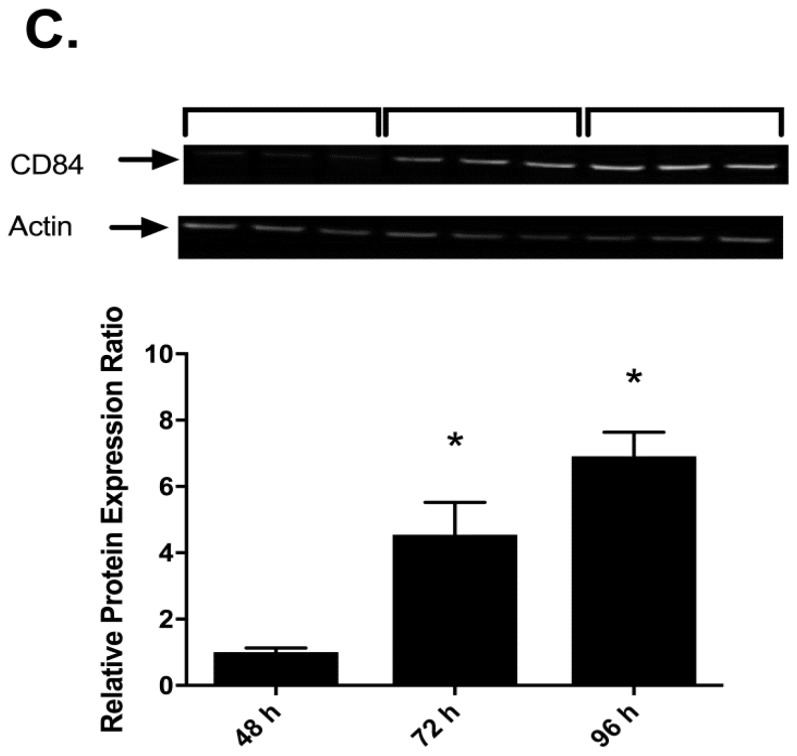
Effects of I3C, DIM and PMA on CD84. (**A**) I3C and DIM on CD84 protein in u-THP-1. THP-1 cells were treated with I3C (5 µM) or DIM (5 µM) for 96 h, cells were harvested for Western analysis and quantitation as described in Materials and Methods. Results expressed as mean ± SD (*n* = 3); (**B**) Effects of PMA on CD84 protein levels. THP-1 cells were differentiated using PMA as described in Materials and Methods. After treatment, cells were harvested for protein and CD84 protein levels were determined by Western blot analysis. Results expressed as mean ± SD (*n* = 3). * indicates significant difference from each other at *p* ≤ 0.05; (**C**) Time-dependent effects of PMA on CD84 protein in THP-1 cells. U-THP-1 cells were treated with PMA for 0–96 h and cells harvested at indicated time points for western analysis and quantitation as described in Materials and Methods. Results expressed as mean ± SD (*n* = 3). * indicates significant difference from 48 h at *p* ≤ 0.05.

To further validate the effects of I3C and DIM on CD84, CD84 protein was determined after THP-1 exposure to I3C and DIM. Treatment of u-THP-1 with I3C or DIM at 5 µM did not change CD84 protein levels (Figure 4A). Even when tested at concentrations as high as 25 µM, no change in CD84 protein levels were observed. 

**Figure 5:**
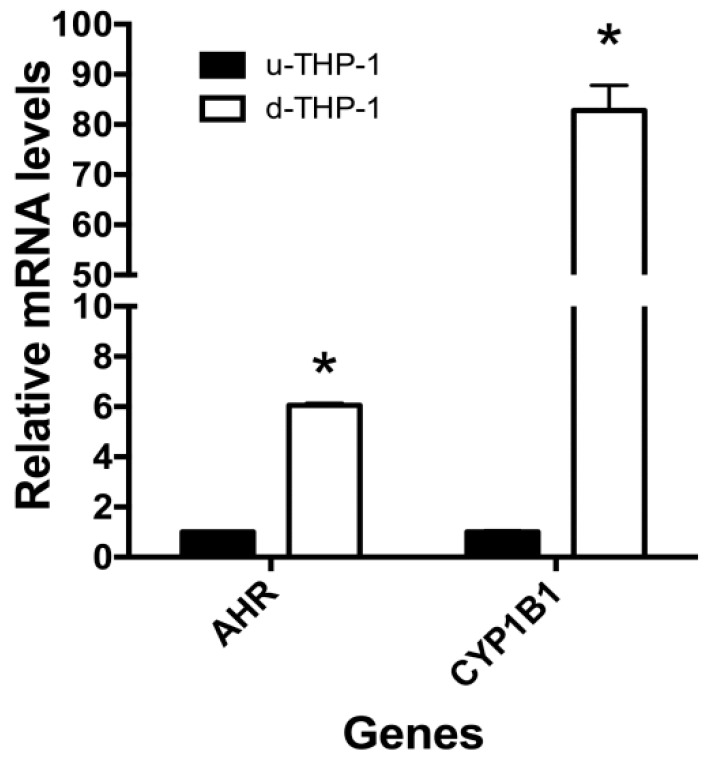
Aryl hydrocarbon (AHR) and CYP1B1 mRNA levels in THP-1 cells. THP-1 cells were differentiated using PMA as described in Materials and Methods. Following treatment, total RNA was isolated and gene expression was determined using RT-PCR. Results are expressed as mean ± SD (*n* = 3) relative to undifferentiated control. * indicates significant difference from undifferentiated control at *p* ≤ 0.05.

However, differentiation appeared to significantly upregulate CD84 mRNA as well as protein levels (Figure 4B,C). PMA induction of u-THP-1 cells to d-THP-1 led to a two to four-fold increase in CD84 mRNA levels (Figure 4B) and significant induction of CD84 protein (Figure 4C). The PMA-induced increase in CD84 protein was time-dependent (Figure 4C). Interestingly, d-THP-1 appeared to express four-fold higher AHR mRNA and 85-fold higher CYP1B1 mRNA than u-THP-1 (Figure 5).

### 2.4. Effects of AHR Agonist beta-Naphthoflavone (b-NF) on CYP1A1/B1, CD84, and LPS-Induced IL-1β mRNA in u-THP-1 Cells

**Figure 6:**
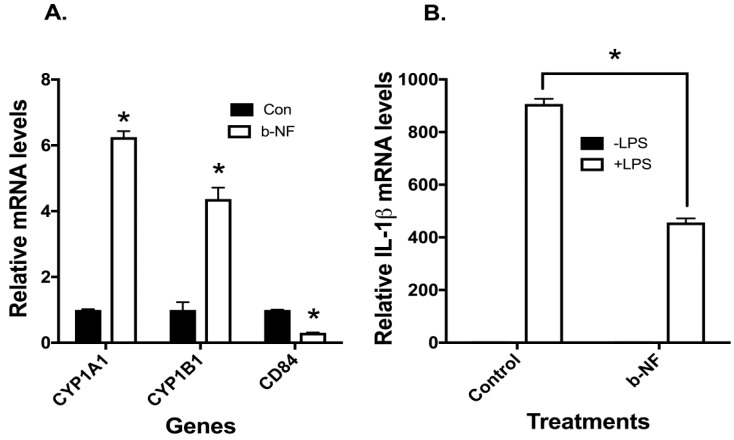
Effects of AHR agonist beta-naphthoflavone (b-NF) on CYP1A1/B1, CD84, and LPS-induced IL-1β mRNA expression in u-THP-1 cells. U-THP-1 cells were treated with or without b-NF (10 µM) as described in Materials and Methods. After treatment with the compounds, cells were exposed to LPS for an additional 2 h. After LPS treatment, total RNA was isolated and CD84, CYP1A1/B1 and IL-1β mRNA expression were determined using RT-PCR. (**A**) Effects on CYP1A1/B1, CD84 expression; (**B**) Effects on LPS induction of IL-1β mRNA expression. Results are expressed as mean ± SD (*n* = 3). * indicates significant difference from vehicle control at *p* ≤ 0.05.

The role of AHR in modulating CD84 and IL1-β was further characterized using the well-documented AHR ligand, b-NF [32]. Exposure of u-THP-1 cells to b-NF (10 µM) led to the induction of CYP1A1/B1 mRNA (Figure 6A). 

Treatment of u-THP-1 cells with b-NF inhibited the expression of CD84 mRNA (Figure 6A) and also decreased LPS-induced IL-1β mRNA levels (Figure 6B). 

### 2.5. Effects of AHR siRNA on I3C, DIM and b-NF-Mediated Changes in IL-1β, CD84 and CYP1B1 mRNA

**Figure 7:**
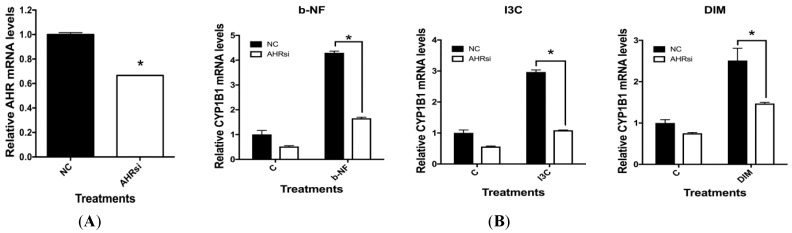

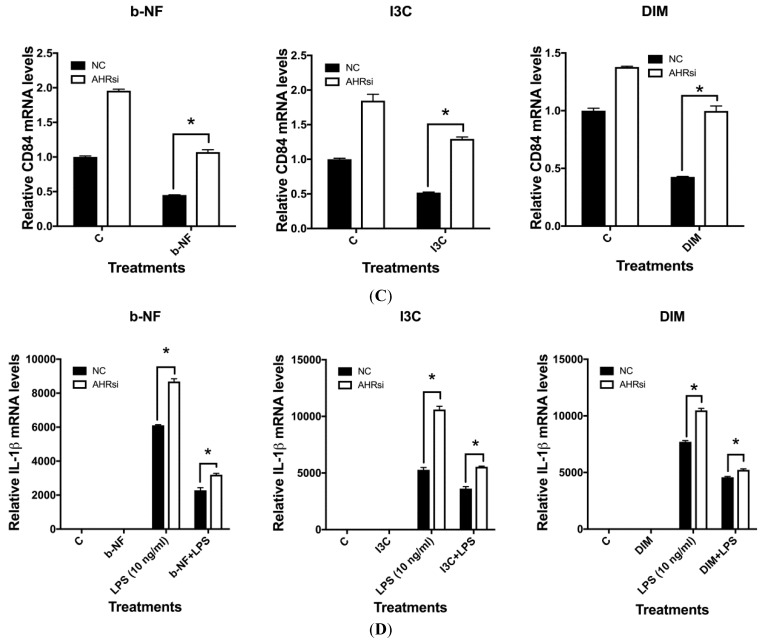
Effect of AHR siRNA on I3C (25 µM), DIM (10 µM) and b-NF (10 µM) modulation of IL-1β, CD84 and CYP1B1 mRNA in u-THP-1 cells. Experiments using AHR siRNA were conducted in u-THP-1 cells as described in Materials and Methods. After treatments, total RNA was isolated and AHR, CD84, CYP1B1 and IL-1β mRNA were determined using RT-PCR. (**A**) Effects of AHR siRNA on AHR mRNA levels; (**B**) Effects of AHR siRNA on CYP1B1 mRNA levels; (**C**) Effects of AHR siRNA on CD84 mRNA levels; (**D**) Effects of AHR siRNA on LPS induction of IL-1β mRNA levels. Results are expressed as mean ± SD (*n* = 3). * indicates significant difference from vehicle control at *p* ≤ 0.05.

Experiments using siRNA against AHR to knockdown AHR expression were performed in order to validate the role and specificity of AHR in I3C and DIM’s modulatory effects on IL-1β and CD84 mRNA. AHR siRNA treatment of u-THP-1 cell led to significant knockdown of AHR mRNA expression (Figure 7A). 

Treatment of u-THP-1 with AHR siRNA also significantly inhibited background expression of CYP1B1 mRNA levels (Figure 7B). Furthermore, AHR siRNA treatment attenuated induction of AHR-dependent CYP1B1 mRNA by I3C (25 µM), DIM (10 µM), and b-NF (10 µM) in u-THP-1 cells. AHR siRNA treatment significantly increased the background expression of CD84 mRNA in THP-1 cells and attenuated the inhibitory effects of I3C, DIM, and b-NF on CD84 mRNA level (Figure 7C). AHR siRNA also attenuated the inhibitory effects of I3C, DIM and b-NF on LPS induction of IL-1β mRNA levels (Figure 7D). 

### 2.6. Effects of I3C, DIM, and b-NF on Other LPS-Inducible Cytokines

**Figure 8:**
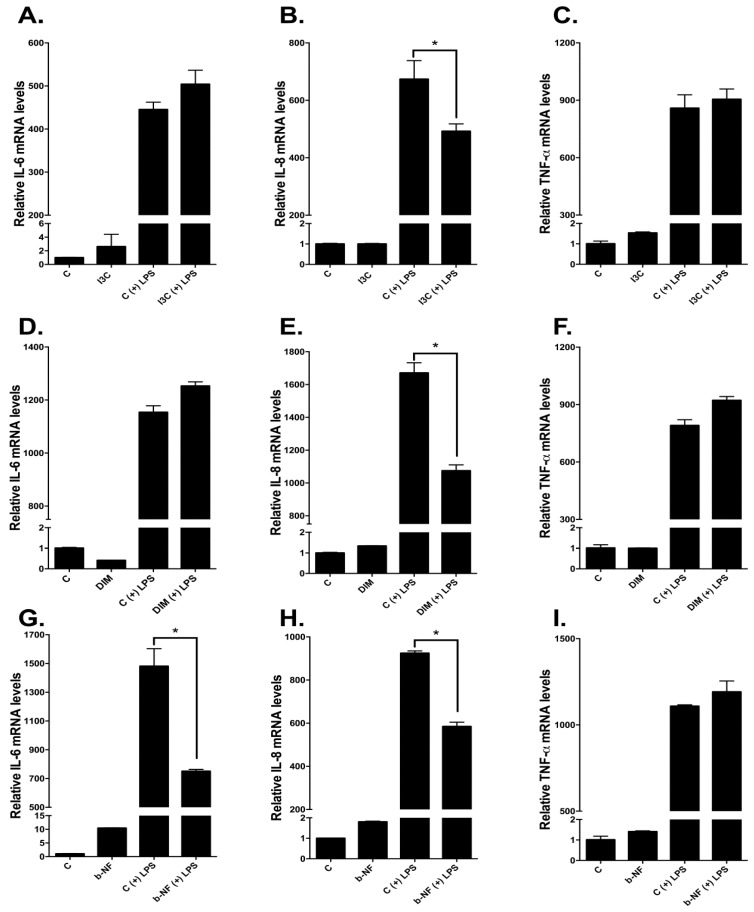
Effects of I3C, DIM and b-NF on LPS-inducible cytokines IL-6, IL-8 and TNF-α mRNA levels in u-THP-1 cells. U-THP-1 cells were treated with or without I3C (25 µM), DIM (10 µM) or b-NF (10 µM) as described in Materials and Methods. After treatment with the compounds, cells were exposed to LPS for an additional 2 h. After LPS treatment, total RNA was isolated and IL-6, IL-8 and TNF-α mRNA expression were determined using RT-PCR. (**A**–**C**): Effects of I3C on IL-6 (**A**), IL-8 (**B**) and TNF-α (**C**); (**D**–**F**): Effects of DIM on IL-6 (**D**), IL-8 (**E**) and TNF-α (**F**); (**G**–**I**): Effects of b-NF on IL-6 (**G**), IL-8 (**H**) and TNF-α (**I**). Results are expressed as mean ± SD (*n* = 3). * indicates significant difference from vehicle control at *p* ≤ 0.05.

We also examined whether other LPS-responsive cytokine genes in u-THP-1 cells are affected similarly to IL-1β by I3C and DIM. Treatment of u-THP-1 cells with I3C (25 µM), DIM (10 µM), and b-NF (10 µM) inhibited LPS induction of IL-8 mRNA levels (Figure 8). By contrast, LPS induction of TNF-α mRNA in u-THP-1 cells was not affected by any of the compounds tested. Interestingly, in u-THP-1 cells, b-NF but not I3C and DIM inhibited LPS induction of IL-6 mRNA levels. 

## 3. Discussion

The current study focuses on addressing two main questions: (1) How do I3C and DIM affect the tumor environment? (2) What is the role of CD84 and AHR in mediating I3C and DIM’s effects? Our results support the modulation of infection/inflammation-related pathways in monocytes but not macrophages as a potential mechanism. Furthermore, AHR but not CD84 appeared to mediate the effects of I3C and DIM in monocytes. Unexpectedly, we also found a novel observation that regulation of CD84 by I3C and DIM appeared to be disconnected between transcription and translational levels in monocytes. 

We tested the effects of the food-derived phytochemicals I3C and DIM on the tumor environment using a cell culture model. Here we reported a novel inhibitory effect of the brassica-derived compounds I3C and DIM on LPS-induced production of IL-1β in monocytes but not in macrophages. We took advantage of the human THP-1 cells’ ability to exist as monocytes (u-THP-1) and macrophages (d-THP-1) to examine the effects of I3C and DIM on cytokines stimulated by differentiation and the TLR ligand. In the u-THP-1 cell, both I3C and DIM significantly inhibited LPS induction of IL-1β at mRNA and protein levels. In both cases, I3C and DIM exerted their effects at physiologically achievable concentrations of 5 and 1 µM, respectively (Figure 1 and Figure 2). We found AHR knockdown attenuated the inhibitory effects of I3C, DIM and the well documented AHR ligand, b-NF, on IL-1β mRNA. Similarly, AHR knockdown also inhibited induction of CYP1B1 mRNA by I3C, DIM and b-NF. Hence, AHR may play a central role in diet-derived AHR ligands, such as I3C and DIM, in regulating responses to inflammation. IL-1β is known to be a pro-inflammatory cytokine that promotes angiogenesis [12,13]. The inhibition of IL-1β by I3C and DIM may lead to the attenuation of tumor angiogenesis and protection against carcinogenesis but warrants further investigation. 

The effects of I3C and DIM appeared to be relatively specific to u-THP-1. In the d-THP-1 cells, I3C and DIM inhibition of LPS-induction of IL-1β was attenuated. This lack of an effect from I3C and DIM may be due to d-THP-1 cell expressing higher baseline levels of both AHR (~4×) and CYP1B1 (~85×) than the u-THP-1 (Figure 5). The higher CYP1B1 levels in d-THP-1 could degrade these compounds and prevent these compounds from interacting with AHR to modulate IL-1β expression. Therefore, we consider monocytes to be a more relevant physiological target than macrophages for I3C and DIM. Given that many phytochemicals also act through AHR pathways [33,34]; attention to these pathways in monocyte vs. macrophage may be necessary in studying the biological efficacies of other AHR-dependent phytochemicals.

We initially hypothesized that the SLAM family protein CD84 may mediate the effects of I3C and DIM on IL-1β. However, our data suggest otherwise. Based on in-silico analysis, several AHR consensus binding sites in the 5′-promoter region (~3000 bp form ATG) of the CD84 gene were identified [31]. AHR siRNA reversed the inhibitory effects by I3C, DIM, and b-NF on CD84 mRNA, confirming that CD84 is indeed an AHR responsive gene. However, we found that—while CD84 mRNA was affected by I3C and DIM in u-THP-1 cells—CD84 protein levels remain unchanged in I3C- or DIM-treated u-THP-1 cells. There appeared to be a disconnect between transcriptional and translational regulation of CD84 in this situation. The lack of CD84 protein changes in u-THP-1 led us to conclude that the inhibitory effect of I3C and DIM on the LPS-induced increase of IL-β was not mediated indirectly through a CD84-related pathway but directly through an AHR pathway. By contrast, PMA-differentiation of THP-1 led to higher levels of CD84 mRNA and this change was reflected at the protein level by Western analysis. These results suggest that CD84 is regulated during differentiation of monocyte to macrophage at the transcriptional level, and the lack of CD84 protein changes in I3C/DIM experiments were not a treatment/detection problem. However, the puzzling question is why there was an increase in CD84 message level in cells treated with I3C or DIM but not in protein level. One possible explanation may be that RT-PCR detection of the message changes may be more sensitive than Western; therefore we observed changes at message level and not at protein level. Also, the CD84 mRNA changes may be short lived and not sufficient to elicit protein changes. Additional studies are warranted to elucidate the significance of this observation. 

Even though all three compounds tested were AHR ligands—based on their abilities to upregulate AHR-responsive genes CYP1A1 and B1—they seemed to act on LPS-induced cytokines differently. None of the test compounds affect TNF-α mRNA levels. By contrast, all compounds inhibited LPS induction of IL-8 mRNA. Only b-NF inhibited LPS induction of IL-6 mRNA. These data suggest that these compounds, in addition to AHR pathways, may act through other mechanisms. Given that both IL-1β and IL-8 are known to promote angiogenesis [12,13,35,36], the inhibitory effect of I3C and DIM on these two cytokines would suggest a regulatory effect of I3C and DIM on the tumor environment that involves angiogenesis. However, the specificity of other diet-derived AHR ligands on cytokines may not be generalized.

**Figure 9:**
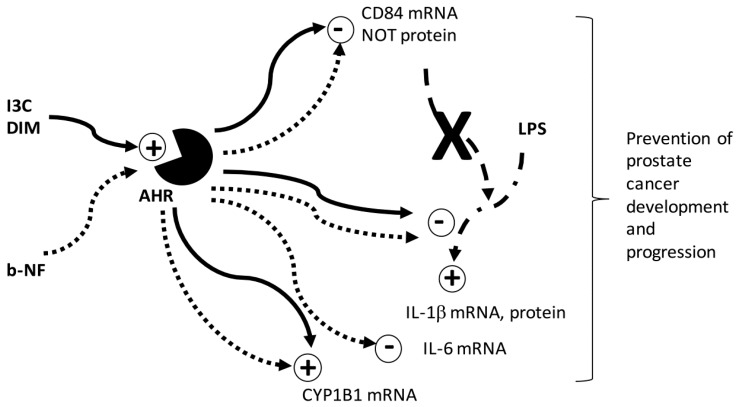
Working Model for regulation of prostate cancer. Schematic diagram shows the proposed mechanism underlying the inhibitory effects of I3C and DIM on LPS induction of IL-1β mRNA and protein, mediated through AHR, and not the CD84-mediated pathway. I3C and DIM down-regulated CD84 mRNA, but not protein levels in an AHR-dependent manner. Activation of AHR is indicated by upregulation of CYP1B1, a well-documented AHR-responsive gene. The classic AHR ligand, b-NF, exerts similar effects as I3C/DIM on IL-1β, CD84, and CYP1B1 mRNA, but only b-NF inhibited LPS-induction of IL-6 mRNA levels. Solid line: Effects of I3C/DIM. Dotted line: Effects of b-NF. Bold X: Based on our data, the pathway does not occur. DIM is known to be an acid condensation product of I3C. The effectiveness of I3C and DIM on the pathway examined is best differentiated by dose dependence of the compounds. Specifically, DIM elicits an effect at a much lower concentration than I3C. However, our results indicate that both I3C and DIM can influence the AHR pathway. Hence, I3C and DIM may directly influence immune cells and also modulate cancer cell-stromal cell interaction to exert their cancer protective effects. The overall effect would reduce monocyte/macrophage infiltration, inflammation and potential for angiogenesis in the prostatic tumor. Further investigations are necessary to validate this hypothesis in vivo in prostate cancer.

Despite extensive research, the precise mechanisms by which I3C and DIM exert their cancer protective effect remain unclear. Our studies suggest that tumor microenvironment may be a potential mechanism by which I3C and DIM exert their effects. However, several mechanisms, acting through both direct and indirect regulation of multiple pathways, may be involved within the tumor microenvironment to promote cancer formation and progression. As we reported earlier [31], I3C and DIM modulation of androgen receptor mediated pathways can attenuate monocyte migration to tumor cells through CCL2-dependent pathways. In the present study, we found I3C and DIM act through AHR to modulate LPS-induction of IL-1β and CD84 (Figure 9). 

## 4. Materials and Methods

### 4.1. Cell Culture

Human monocytic THP-1 cells were purchased from the American Type Culture Collection (Manassas, VA, USA) and grown in RPMI 1640 media supplemented with 100 U/mL penicillin, 100 µg/mL streptomycin (Pen/Strep), and 10% fetal bovine serum (FBS) (Invitrogen, Carlsbad, CA, USA). Cells were incubated at 37 °C in the presence of 5% CO_2_. All experiments were conducted in the same culture media.

### 4.2. THP-1 Cell Differentiation

Human THP-1 cells were differentiated to macrophage phenotype using phorbol 12-myristate 13-acetate (PMA) (Sigma, St. Louis, MO, USA). Undifferentiated THP-1 cells (u-THP-1 or monocytes) were plated in 6-well plates (Costar, Corning Incorporated, Corning, NY, USA) at a concentration of 2.5 × 10^5^ cells/mL in 2 mL media containing 25 ng/mL PMA. The plates were wrapped with aluminum foil, as PMA is light sensitive, and incubated at 37 °C/5% CO_2_ for 48 h. After incubation, the differentiated THP-1 cells (d-THP-1), now attached onto the plate, were then washed once with room temperature PBS before starting treatment.

### 4.3. Cell Treatments

U-THP-1 or d-THP-1 cells were cultured in 6-well plates in the culture medium described above. DIM (Sigma, St. Louis, MO, USA) (0, 1, 5, 10 or 25 µM) or I3C (Sigma, St. Louis, MO, USA) (0, 5, 25 or 50 µM) was added to the media at indicated concentrations; fresh media containing the compounds was replaced every 24 h. Ethanol was used as vehicle control. After 48 h incubation, u-THP-1 cells were collected by centrifugation (400 RCF for 5 min) for RNA isolation. For d-THP-1, cells were lysed directly with 1 mL TRIzol reagent (Life Technologies, Carlsbad, CA, USA) added onto 6-well plates for RNA isolation as described below. For gene expression experiments using LPS, after 48 h of treatment with the compounds, cells were then treated with or without 10 ng/mL LPS (Sigma, St. Louis, MO, USA) for an additional 2 h and harvested as described above.

### 4.4. Total RNA Isolation, Reverse Transcription (RT)-PCR and Gene Expression Analysis

Total RNA Isolation, Reverse Transcription (RT)-PCR and gene expression analyses were conducted as previously described [24]. Briefly, cells were washed with PBS once and TRIzol reagent was added for total RNA isolation as described previously [24]. Affinity Script Multiple Temperature cDNA Synthesis kit (Agilent Technologies, Santa Clara, CA, USA) was used for cDNA synthesis, and 1 µg of total RNA was used to reverse-transcribe mRNA to cDNA. Real-time PCR was performed on ViiA7 Real-Time PCR Detection System using the TaqMan Universal Fast Master Mix according to the manufacturer’s protocol, and 5 µL of cDNA was used for real-time PCR. TaqMan gene expression assay (Life Technologies, Carlsbad, CA, USA) was used to quantify gene expression levels of human AHR (Hs00169233_m1), CYP1A1 (Hs00153120_m1)/B 1 (Hs00164383_m1), CD84 (Hs01547121_m1), IL-1β (Hs01555410_m1), IL-6 (Hs00985639_m1), IL-8 (Hs00174103_m1) and TNF-α (Hs00174128_m1). Human TATA box binding protein (TBP) (Hs00427620_m1) was used as a housekeeping gene for calculation of relative expression levels using the ΔΔ*C*_t_ method as previously described [24]. 

### 4.5. Determining Effects of I3C and DIM on IL-1β Protein Secretion by THP-1

THP-1 monocytes were seeded at 2.5 × 10^5^ cells/mL in a 6-well plate. After 24 h incubation, cells were treated without or with test compounds for 48 h with a media change every 24 h. After 48 h of treatment, cells were then treated with or without 10 ng/mL LPS (Sigma, St. Louis, MO, USA) for an additional 6 h. After LPS treatment, cell media were collected by spinning down cells in 15-mL tubes at 1000 RCF for 10 min. Cell pellets were washed once with PBS and re-suspended in 100 µL of RIPA buffer (Pierce, Rockford, IL, USA) containing 5 mM EDTA and protease inhibitor (1× Halt™ protease inhibitor cocktail, Pierce, Rockford, IL, USA). Both media and cell lysates were then stored at −80 °C for total protein and IL-1β determination. The Quantikine ELISA-Human IL-1β/IL-1F2 Immunoassay kit (R&D systems, Minneapolis, MN, USA) was used to measure IL-1β protein level secreted into media according to the manufacturer’s protocol. Protein concentrations of the cell pellets were determined using the BCA method according to the manufacturer’s protocol (Thermo Scientific, Rockford, IL, USA). The amount of IL-1β in the media was normalized to the respective cell protein concentration and expressed as pg/mL. 

### 4.6. Effects of AHR siRNA on I3C, DIM and β-Naphthoflavone-Mediated Changes

THP-1 monocytes were seeded at 2.5 × 10^5^ cells/mL in a 6-well plate. SMARTpool ON-TARGET AHR siRNA or ON-TARGET plus Non-target siRNA (GE Healthcare Dharmacon, Lafayette, CO, USA) and HiPerFect Transfection Reagent (Qiagen, Louisville, KY, USA) were used for AHR knockdown experiments according to manufacturer’s protocol. Briefly, siRNA/HiPerFect complex was made and added dropwise to cell pellets from cells seeded 24 h prior. The whole cell/complexes solutions, in 15-mL tubes, were incubated for 6 h at 37 °C, 5% CO_2_ incubator before transferring to a 6-well plate. Culture medium in the presence of 10% FBS and 1% Pen/Strep was added. Plates were then incubated for 48 h in the incubator before phytochemicals were added for the next 48 h. Media were changed every 24 h, followed by the addition of LPS and incubated for another 2 h before RNA was isolated as previously described. The effects of knockdown were assessed by determining mRNA levels of AHR and the LPS-responsive genes IL-1β.

### 4.7. Western Blot Analysis of CD84 Protein Levels

The protein levels of CD84 were assessed using Western blot analysis. The u-THP-1 cells (2.5 × 10^5^ cells/mL) were treated with or without 5 µM I3C or DIM in T-175 cm^2^ flasks for 96 h. Cells were collected by centrifugation at 400 RCF for 5 min, media was removed by aspiration, and cell pellets washed once with 15 mL of phosphate-buffered saline (PBS) and centrifuged again at 400 RCF for 5 min. After centrifugation, PBS was removed by aspiration and cells were lysed in 200 µL RIPA buffer containing EDTA and protease inhibitors. The lysates were homogenized on ice three times (10 s each) using a Branson Digital Sonifier (Branson, Danbury, CT, USA) followed by centrifugation at 10,600 RCF for 15 min at 4 °C. Supernatants were collected and protein concentrations were tested using BCA assay following manufacturer’s protocol (Thermo Scientific, Rockford, IL, USA). For d-THP-1, u-THP-1 cells were treated with 25 ng/mL PMA for 0, 48, 72, and 96 h and then collected by washing with 15 mL of PBS followed by scraping using a Falcon cell scraper (Corning Life Sciences, Acton, MA, USA). The harvested cells were then centrifuged at 400 RCF for 5 min and 200 µL RIPA lysis buffer containing EDTA and protease inhibitors was added to cells after PBS removal. The lysates were treated similarly to u-THP-1 as described above. Supernatants were collected and protein concentrations were determined using the Pierce BCA assay following the manufacturer’s protocol (Thermo Scientific, Rockford, IL, USA). Routinely, 10 µg protein sample per lane was used for electrophoresis separation on SDS-PAGE using 10% Bis-Tris gel following the manufacturer’s protocol (NUPAGE, Invitrogen, Carlsbad, CA, USA). After electrophoresis, the protein was transferred from the gel onto a nitrocellulose membrane using iBlot (Invitrogen, Carlsbad, CA, USA) apparatus according to the manufacturer’s procedure. After transfer, the nitrocellulose membrane was then blocked in 1× TBS buffer (Thermo Scientific) containing 5% milk for 1 h at room temperature while shaking, followed by washing with SuperBlock (TBS) T20 (Thermo Scientific) blocking buffer (3 times, 5 min each). After washing, the membrane was then incubated with mouse monoclonal anti-CD84 (sc-23899, Santa Cruz Biotechnology, Dallas, TX, USA, 1:100 dilution) primary antibody or rabbit polyclonal actin antibody (sc-1616-R, Santa Cruz Biotechnology, 1:20,000 dilution) in blocking solution overnight at 4 °C. After overnight incubation, the membrane was washed with 1X TBS Tween 20 buffer (3 times, 5 min each) and incubated with the IRDye 800 CW goat anti-mouse or goat anti-rabbit (LiCOR, Lincoln, NE, USA) secondary antibody (1:20,000 dilution) in SuperBlock T20 for 2 h, in the dark, at room temperature. After the secondary antibody incubation, the membrane was washed with 1× TBS Tween 20 buffer and preserved in 1× TBS buffer before imaging and quantitation. Specific CD84 and actin proteins were detected and quantified using the LICOR ODYSSEY^®^ CLx (LiCOR, Lincoln, NE, USA) Infrared Imager according to manufacturer’s procedure.

### 4.8. Statistical Analysis

All experiments were performed in triplicate, and data were reported as the mean ± standard deviation (SD). GraphPad Prism for Windows (Prism 7, GraphPad Software Inc., La Jolla, CA, USA) was used for statistical analysis. Depending on the experimental design, multiple group experiments were analyzed using one- or two-way ANOVA followed by post-hoc test. *p* values ≤ 0.05 were considered significant.

## Abbreviations

AHR	aryl hydrocarbon
b-NF	beta-naphthoflavone
CCL2/MCP-1	C-C Motif Chemokine Ligand 2/Monocyte Chemoattractant Protein-1
DIM	3,3′-diindolylmethane
u-THP-1	undifferentiated THP-1 cells
d-THP-1	differentiated THP-1 cells
I3C	indole-3-carbinol
IL-1β	interleukin-1β
LPS	lipopolysaccharide
PMA	phorbol 12-myristate 13-acetate
SLAM	signaling lymphocyte activation molecule
TLR	toll-like receptor
TNF-α	tumor necrosis factor-α
